# RNA binding protein BOULE forms aggregates in mammalian testis

**DOI:** 10.7555/JBR.36.20220072

**Published:** 2022-06-28

**Authors:** Yujuan Su, Xinghui Guo, Min Zang, Zhengyao Xie, Tingting Zhao, Eugene Yujun Xu

**Affiliations:** 1 State Key Laboratory of Reproductive Medicine, Nanjing Medical University, Nanjing, Jiangsu 211166, China; 2 Department of Neurology, and Center for Reproductive Sciences, Northwestern University, Chicago, IL 60611, USA

**Keywords:** amyloid, RNA-binding protein, BOULE, protein aggregation, SDD-AGE, enhanced UV cross-linking immunoprecipitation

## Abstract

Amyloids have traditionally been considered pathologic protein aggregates which contribute to neurodegeneration. New evidence however increasingly suggests that non-pathological amyloids are formed in animals during normal development. Amyloid-like aggregate formation was originally thought to be a conserved feature of animal gametogenesis. This hypothesis was based on findings which suggest that regulated amyloid formations govern yeast meiosis by way of meiosis-specific RNA binding proteins. Additional support came from studies which demonstrate that DAZL, a mammalian gametogenesis-specific RNA binding protein, also forms SDS-resistant aggregates *in vivo*. Here, we report evidence of aggregated BOULE formations, another DAZ family protein, during sperm development. Data suggest that in mouse testis, BOULE forms SDS-resistant amyloid-like aggregates. BOULE aggregate formation correlates with dynamic developmental expression during spermatogenesis but disappeared in *Boule* knockout testis. We also mapped essential small region *in vitro* BOULE aggregations, immediately downstream DAZ repeats, and found that aggregations positively correlated with temperature. We also performed enhanced UV cross-linking immunoprecipitation on BOULE aggregates from mouse testes and found that aggregates bind with a large number of spermatogenesis-related mRNAs. These findings provide insight into the amyloidogenic properties of gametogenesis-specific RNA binding proteins as a conserved feature in mammalian reproduction. Further investigation is warranted to understand the functional significance of BOULE amyloid-like formation during mouse spermatogenesis.

## Introduction

Recent studies have shown that intracellular RNA-binding proteins (RBP) can play a role in neuronal development and through gametogenesis^[1–2]^, as well as during other processes. This is because RBPs transform their physical state in order to generate functional aggregates^[3–6]^. RNA binding proteins in germ cells are also known to form membrane-less granules, also known as liquid droplets or condensates, in phase transitioning^[7]^. While physiological requirements and the function of human fertility factors are generally understood using mouse models^[8]^, little is actually known about how physical states and state transitions of those factors contribute to mammalian gametogenesis.

In yeast, the RNA-binding protein Rim4 mediates translational repression of numerous mRNAs by assembling them into amyloids. This shows that amyloid can be utilized as central regulators of gametogenesis and further, deletion of the low-complexity region prevents Rim4 amyloid formation and its ability to repress translation^[1,9]^. Similarly, Rim4, which is a member of the mammalian Deleted in Azoospermia (DAZ) protein family, is also a gametogenesis-specific RNA binding protein containing RNA recognition motif. The human DAZ protein family consists of Y-linked DAZ genes and autosomal homologs BOULE and DAZL, all of which function only in the testis or ovaries^[10–12]^. Human BOULE and DAZ proteins have a propensity to aggregate when expressed in yeast, and DAZ proteins are thought to contain a prion-like domain^[13–14]^. In addition, mouse DAZL can also form aggregates in mouse testis^[1]^; however, DAZ family protein aggregate-formation in mammalian spermatogenesis has not been explored. Given that BOULE is the oldest member of the DAZ family and is functionally conserved from fly to human^[10,15]^, we hypothesize that conserved BOULE might aggregate. Therefore, we chose BOULE to examine whether DAZ family proteins undergo amyloid-like aggregation during spermatogenesis. Such characterization may provide insights into mechanisms involved in DAZ protein regulation in mammalian fertility.

BOULE is conserved in sequences in the metazoan animal world and from sea anemones to humans. Mouse and fly *Boule* homologs also share a conserved spermatogenic requirement^[10,15]^. In a previous study, we found that *Boule* genes are lacking in mice which leads to male-specific infertility with sperm development arrested at the spermatid stage^[10,16]^. However, the specific mechanism involved in BOULE spermatogenesis regulation remains unclear. Based on the hypothesis that amyloid-like protein aggregation is a conserved feature of animal gametogenesis^[1]^, we proposed to determine whether BOULE could form amyloid-like aggregates during spermatogenesis in mice. We also looked to investigate BOULE functions through this evolutionarily conserved form as a functional amyloid-like aggregate.

Through the *in vivo* and the *in vitro* studies of mouse BOULE protein, we interrogated the aggregation propensity of mammalian BOULE protein, identified the regions essential for aggregation and established the functional significance of BOULE aggregation by performing eCLIP on aggregated BOULE protein in the testis. Our data support that BOULE aggregation occurs during spermatogenesis as such their functional significance during mammalian spermatogenesis warrants further investigation.

## Materials and methods

### Animals

Wild type and *Boule* knockout mice were raised under standard conditions in the animal facilities of Nanjing Medical University, Nanjing, China.Protocols were approved by the Institutional Animal Care and Use Committees (IACUC) of Nanjing Medical University. All procedures were conducted according to institutional guidelines for the care and use of animals. Mice with a mixed 129/B6 background were back-crossed to C57/B6 over at least twenty generations with no changes in spermatogenic defects observed. All wild-type (WT) tissues, heterozygotes or homozygotes were from mice with mixed backgrounds from the same litter.

### Isolation of spermatogenic cells

Pachytene spermatocytes, round and elongated spermatids were isolated from adult mice using the STA-PUT method^[17]^ with minor modifications. Testes were harvested and digested using collagenase type Ⅳ at 1 mg/mL. The dispersed seminiferous tubules were washed using Dulbecco's modified Eagle's medium (DMEM) (Gibco, USA) and centrifuged at 500 *g*. The pellet was then digested with 0.25% Trypsin (Gibco) containing DNase Ⅰ (1 mg/mL; Sigma, USA) and filtered to prepare a single-cell suspension. Then, the single-cell suspension was loaded into a cell separation apparatus (ProScience, Canada), following a 2% to 4% bovine serum albumin (BEST, China) gradient. After 1.5 to 3 hours of sedimentation, cell fractions were harvested. The purity of different isolated cell types was determined according to morphological characteristics, cell diameter, and DAPI (Sigma) staining under a light microscope. The purity of spermatogonia, pachytene spermatocytes, and round spermatids is approximately 90%.

### Semi-denaturing detergent agarose gel electrophoresis assay

Semi-denaturing detergent agarose gel electrophoresis (SDD-AGE) was adapted from previous protocols described by Halfmann *et al,* with minor modifications^[18–19]^. Testis proteins and purified BOULE proteins were harvested and lysed in lysis buffer (50 mmol/L Tris-HCl, pH 7.5, 150 mmol/L NaCl, 0.5% Triton X-100, 10% glycerol with 1× protease inhibitor cocktail [Roche, Switzerland]) for 30 minutes. Lysates were clarified twice through centrifugation at 5000 *g* for 5 minutes at 4 °C. 4× Sample loading buffer (2× TAE, 20% glycerol, 8% SDS, bromophenol blue) was added to lysates which were incubated for 10 minutes at room temperature, followed by loading with samples on newly-prepared 1.5% agarose gel with 0.1% SDS and electrophoresis in running buffer (1× TAE and 0.1% SDS) for 10 hours with a constant voltage of 29 V at 4 °C. Proteins were then transferred to the nitrocellulose filter membrane filter membrane (Merck Millipore, USA) for Western blotting with indicated antibodies by capillary action.

#### Separation of Seprion PAD-beads

The Seprion PAD-beads (SEP) isolation method is based on the proprietary ligand effect for retaining protein aggregates. A PAD-Bead kit (Microsens Biotechnologies, UK) was purchased and samples were treated in accordance with the manufacturer's protocol. Firstly, 25 mg of mouse testis tissue was homogenized in a total volume of 200 µL 10% sucrose. Then SDS was added to the homogenate to a final concentration of 0.1% (w/v) to enable solubilization. The captured protein was washed following the manufacturer's protocol and eluted at 95 °C for 5 minutes.

### Western blotting

Tissues or cell samples were prepared by RIPA (Beyotime, China) and protease inhibitor cocktail. Aliquots of the lysates were electrophoresed in 10% polyacrylamide gel and transferred to polyvinylidene difluoride membrane (Bio-Rad, USA). The membrane was blocked with 5% skim milk/TBST (Tris-buffered saline with 0.1% Tween-20) and probed with various antibodies, *e.g.*, rabbit anti-BOULE (1:1000 dilution; Boule anti-serum 101)^[16]^, rabbit anti-PUM2 antibody (1:1000 dilution; Cat. No. ab10361, Abcam, USA); rabbit anti-PABP antibody (1:1000 dilution; Cat. No. ab21060, Abcam); rabbit anti-α-tubulin antibody (1:5000 dilution; Cat. No. SC-8035, Santa Cruz Biotechnology, USA); anti-β-actin (1:1000 dilution; Cat. No. SC-1615, Santa Cruz Biotechnology); mouse anti-FLAG antibody (1:2000 dilution; Cat. No. abF1804, Sigma). Detection of HRP conjugated secondary antibody was performed with enhanced chemiluminescence detection reagents (ECL Kit; PerkinElmer, USA).

### Quantitative real-time PCR

For quantitative real-time PCR (qPCR), the RNA was converted to cDNA with random primers (Takara, Japan). qPCR was performed using a SYBR Green Master Mix Kit (Vazyme Biotech). Relative gene expression was analyzed based on the 2^−ΔΔCt^ method with *Gapdh* as internal control. At least three independent experiments were analyzed. All primers were listed in the ***Supplementary Table 1*** (available online).

### Immunofluorescence

The entire testes were fixed with Hartman's Fixative (Sigma), then paraffin-embedded and serially sectioned (5 μm) according to standard protocols^[11,16]^. Sections were used to perform immunofluorescence analysis. The primary antibodies used were as follows: rabbit anti-amyloid fibrils OC (1:1000 dilution; Cat. No. ab2286, Merch Millipore); rabbit anti-amyloid oligomer/rabbit anti-A11 (1:1000 dilution; Cat. No. ab9234, Merck Millipore); rabbit anti-BOULE (1:200 dilution; Boule anti-serum 101)^[16]^.

### Expression and purification of recombinant BOULE proteins

The bacterial expression vector pET28a-His6-mBOULE-FL (full-length) was transformed into BL-21. Protein expression was induced with 0.5 mmol/L IPTG (Beyotime) at 16 °C for 20 hours. After sonication in lysis buffer (50 mmol/L Tris-HCl [pH 8.0], 150 mmol/L NaCl, 10% glycerol, 0.1% TritonX-100, 1 mmol/L PMSF, and 10 mmol/L imidazole), cell lysates were centrifuged at 20 000 *g* for 30 minutes. His6-mBOULE-FL in the supernatant were purified using Ni-NTA spin column (Bio-Rad), washed with wash buffer (50 mmol/L Tris [pH 8.0], 150 mmol/L NaCl, 10% glycerol, 0.1% TritonX-100, 1 mmol/L PMSF, and 20 mmol/L imidazole) and eluted with elution buffer (50 mmol/L Tris [pH 8.0], 150 mmol/L NaCl, 10% glycerol, 0.1% TritonX-100, 1 mmol/L PMSF, and 200 mmol/L imidazole), then verified using Coomassie Brilliant Blue staining (***Supplementary Fig. 1***, available online). Fractions containing His6-mBOULE-FL were pooled and loaded onto a Amicon Ultra-4 filtration column (Merck Millipore) equilibrated with lysis buffer (50 mmol/L Tris-Hcl [pH 7.5], 150 mmol/L NaCl, 0.5% TritonX-100, 1× cocktail).

To verify the effect of different temperatures and concentrations on purified BOULE protein, concentrated BOULE solution was serially diluted into 9 mg/mL (280 mmol/L), 0.9 mg/mL (28 mmol/L), 0.09 mg/mL (2.8 mmol/L), the purified protein of each concentration was evenly divided into three parts, and placed at 4 °C, 25 °C and 37 °C for 30 minutes, and then followed by the SDD-AGE assay.

### Plasmid construction and transfection

For BOULE expression using the pCMV6 vector, segments of the mouse BOULE were subcloned into the pCMV6 vector using *Sgf*I and *Mlu*I restriction enzymes (New England BioLabs Inc., USA). pCMV6-mouse BOULE deletion plasmids (Del-1 to Del-5) were generated by PCR amplification of the coding sequences (CDS) followed by recombining into pCMV6-Entry destination plasmid (Origene Inc., USA) using the ClonExpress MultiS One Step Cloning Kit (Vazyme Biotech). HEK293T cells in a 6-well plate were transfected with 5 μg of pCMV6 construct carrying either segment of BOULE using ExFect Transfection Reagent (Vazyme Biotech). After 48 hours, cells were harvested for SDD-AGE analysis according to the manufacturer's instructions.

For BOULE expression using pCDNA3.1, segments of the mouse BOULE were subcloned into the pCDNA3.1 vector by using *Hin*dIII and *Bam*HI restriction enzymes (New England BioLabs Inc.). Plasmid pCDNA3.1-EGFP-mBOULE-FL and pCDNA3.1-EGFP-mBOULE-Delc were generated by PCR amplification of the CDS followed by recombining into pCDNA3.1-Entry destination plasmid (Invitrogen, USA) using the ClonExpress MultiS One Step Cloning Kit. Hela cells in a 3.5 cm glass dish plate were transfected with 4 μg of pCDNA3.1 construct carrying either segment of BOULE using ExFect Transfection Reagent. Droplets were observed 24 hours later on a glass-bottom cell culture dish for fluorescence imaging.

### Fluorescence recovery after photobleaching analysis

EGFP-mBOULE-FL proteins were used to label liquid droplets *in vitro*. We performed fluorescence recovery after photobleaching (FRAP) assaying in HELA cells and FRAP experiments were performed on a confocal microscope (LSM 800, Zeiss) at room temperature. Defined regions were photobleached at 488 nm and the fluorescence intensities in these regions were collected every 1 second for *in vitro* droplets and normalized to the initial intensity before bleaching. Image intensity was measured in the region of interest.

#### BOULE aggregates enhanced UV cross-linking immunoprecipitation

BOULE aggregates enhanced UV cross-linking immunoprecipitation (eCLIP) was performed as previously described^[20]^. This eCLIP protocol started with UV crosslinking of seminiferous tubules from mouse testes on postnatal day (P) 25, followed by partial RNase digestion and immunoprecipitation using an anti-BOULE polyclonal serum. RNA-protein extracts from input, and from BOULE-IP of both wild-type testes and *Boule* knocked-out testes were eluded with an acidic elution buffer (pH 2.0 at 25 °C and later neutralized by pH 9.8 Tris buffer), based on non-denaturing elution (Dynabeads Protein G; Cat. No. 10004D, Thermo Fisher Scientific, USA), and was run by SDD-AGE and transferred to nitrocellulose filter membrane, specific regions (250 kDa or bigger bands) were excised from BOULE-IP lane. Only regions corresponding to aggregated BOULE were excised at molecular weight of 250 kDa and higher while leaving lower molecular weight corresponding to the BOULE monomers untouched. The region from 75 kDa or higher from both the input lane and *Boule* KO lane was then excised for library construction and sequence analysis as background control. This acidic elution step of the beads and SDD-PAGE are the main difference between aggregates eCLIP and regular eCLIP. The excised protein-bound RNAs were treated with proteinase K to extract aggregate-bound RNAs. After reversed transcription, the adapter is ligated to its 5′ end. Then, the index primer was ligated to cDNA followed by PCR amplification. We sequenced a number of subclones to confirm the quality of libraries before high-throughput library sequencing.

### Bioinformatics analysis

#### Sample demultiplexing and trimming

Samples of IP and Input were demultiplexed by inline barcode^[20]^. Then, the 3′ adapters and 5′ adapters of raw reads were trimmed using in-house parameters which run two rounds according to library construction and structures of sequenced reads.

#### Removing repetitive elements and mapping

Trimmed reads were mapped to RepBase (Mouse RepeatMasker, version mm10) using STAR software to control for spurious artifacts from rRNA and other repetitive reads. Then, unmapped reads from the last step were mapped to the Mus musculus genome (UCSC, mm10).

#### Peak calling

After removing the repetitive elements and mapping with the mouse genome, the uniquely mapping reads were followed the algorithm field to remove eCLIP PCR duplicates. Then, we used tag2peak.pl in the CLIP Tool Kit, which calculates the number of overlapping CLIP tags in each genomic position to identify peaks for the next normalization.

#### Normalization with biological input

BOULE eCLIP peaks of two replicates were normalized by 2 corresponding Inputs *via* a script^[20]^ to remove background noise signals. After that, we set different fold change lines to select significant peaks through normalization controls for further analysis.

### Statistical analysis

Statistical analyses were performed using GraphPad Prism 8.3.0 (GraphPad, USA). All quantitative experiments were repeated three times. The data were expressed as mean±SD. Two-group comparisons were performed using two-tailed unpaired *t*-test. The threshold for statistical significance was set at *P*<0.05.

## Results

### BOULE formed SDS-resistant aggregates in the testis

**Figure 1:**
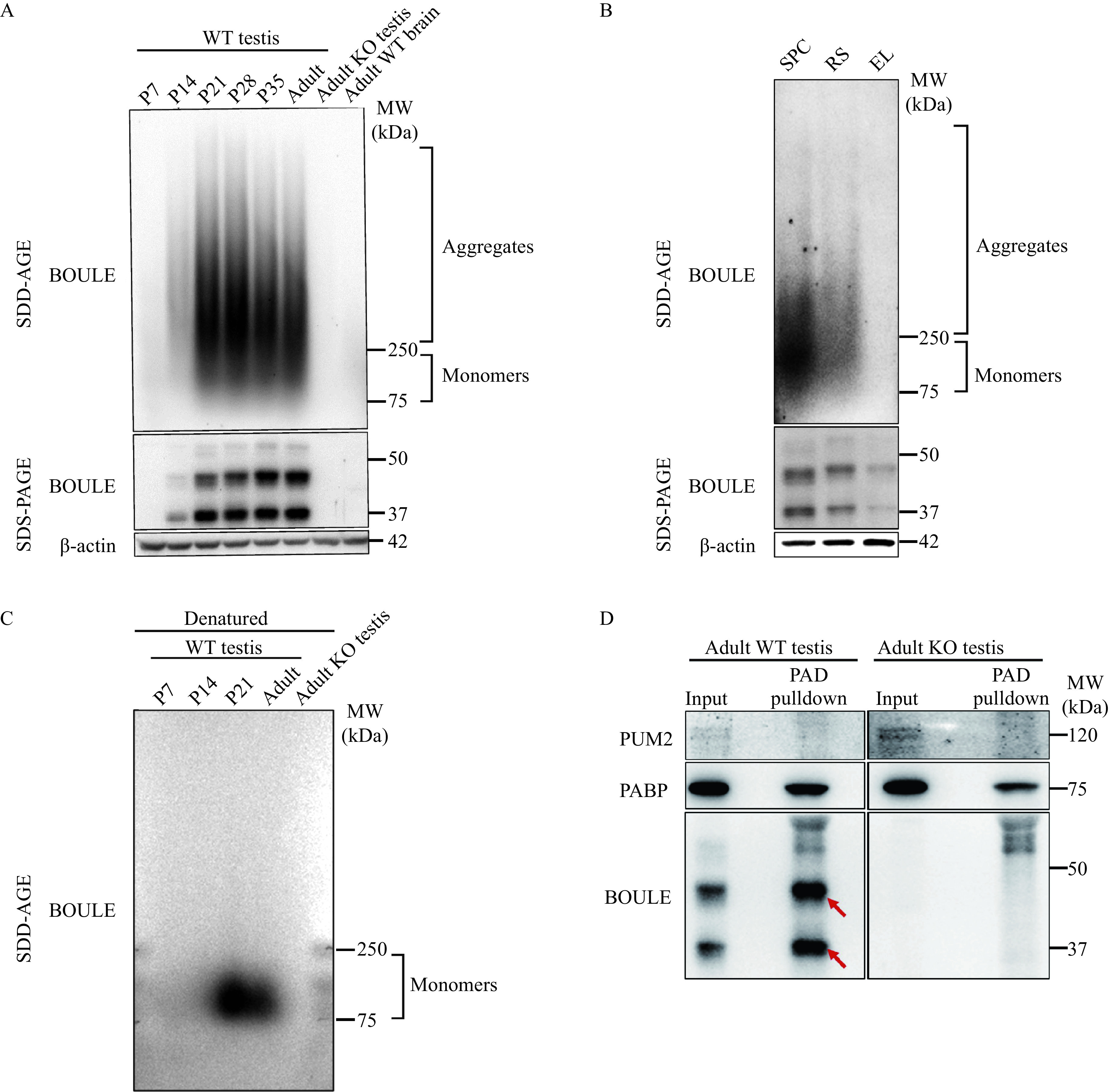
Mouse BOULE formed SDS-resistant aggregates in the testis.

Since DAZL can form aggregates^[1]^, we asked whether the DAZL paralog, BOULE protein can also form aggregates. Many neurodegenerative-disease proteins and yeast proteins form fibrillar amyloids which can be seen using SDD-AGE^[2,9,18,21–22]^. Using this same method, we found a smear of SDS-resistant high-molecular-weight BOULE aggregates which were higher than 250 kDa in mice testis, and several times larger than the predicted 37 kDa, 45 kDa, and 55 kDa of BOULE monomer (***Fig. 1A***).

Given that murine BOULE was expressed in spermatogenic cells from pachytene to round spermatids^[12]^, we asked at what stage of spermatogenesis BOULE aggregates could be detected. Taking advantage of the synchronous spermatogenic development during the first wave of spermatogenesis in neonatal mice, we examined the expression of BOULE aggregates in the testes from P7, P14, P21, P35, and in adult mice, BOULE aggregates were detected first in P14 testis at a very low level. However, this increased significantly in P21 testes and persisted through and into adult testis (***Fig. 1A***). In P14 testis, spermatogenesis reached the early pachytene stage, suggesting that BOULE aggregate expression starts in early pachytene spermatocytes. A high molecular weight smear, detected using the BOULE antibody, was absent in the testis of *Boule* knockout mice^[16]^ and wild-type brain tissue where there is no BOULE expression (***Fig. 1A***). This appears to confirm that these aggregates are specific to BOULE proteins.

We also performed standard Western blotting analysis of proteins extracted from the same testes using SDS-PAGE to detect BOULE monomers. BOULE aggregates detected using SDD-AGE and BOULE monomers detected by SDS-PAGE exhibited similar expression dynamics, low at P14 and high from P21 to adult (***Fig. 1A***). Concordant expression between BOULE monomers and aggregates was also observed in purified spermatogenic cells under the STA-PUT method. This supports the notion that BOULE aggregates are expressed specifically in spermatocytes and in round spermatids (***Fig. 1B***). A major difference between SDD-AGE and SDS-PAGE was the sample preparation, samples for SDD-AGE were not heat-denatured and there was no reducing agent (β-mercaptoethanol) in SDD-AGE lysis buffer. We prepared mouse testis protein extract samples by adding β-mercaptoethanol and denaturation them by heating, which resulted in complete denaturation of protein aggregates. We found that only BOULE protein signals at sizes smaller than 250 kDa but bigger than 75 kDa were detected by SDD-AGE gel, representing BOULE monomers on SDD-AGE gel (***Fig. 1C***). This experiment further supports the hypothesis that high molecular weight BOULE aggregates under SDD-AGE are indeed aggregates of many BOULE monomers.

### BOULE aggregates formation was confirmed by PAD assay and co-localized with fibril proteins during sperm development

To further verify the existence of BOULE aggregates, we used a commercially available protein aggregate binder *i.e.*, SEP on the testis BOULE aggregates^[23]^. We found BOULE proteins are enriched by SEP from wild type testes but not in the testis of *Boule* KO mice. This further supports the idea that BOULE can form aggregates (***Fig. 1D***). PAD is widely used in commercial settings and amyloid disease diagnoses such as bovine spongiform encephalopathy (aka mad cow disease) by pulling down cross beta-sheet amyloid. The successful pull-down of BOULE protein aggregates by PAD supported BOULE could therefore form amyloid-like aggregates.

**Figure 2:**
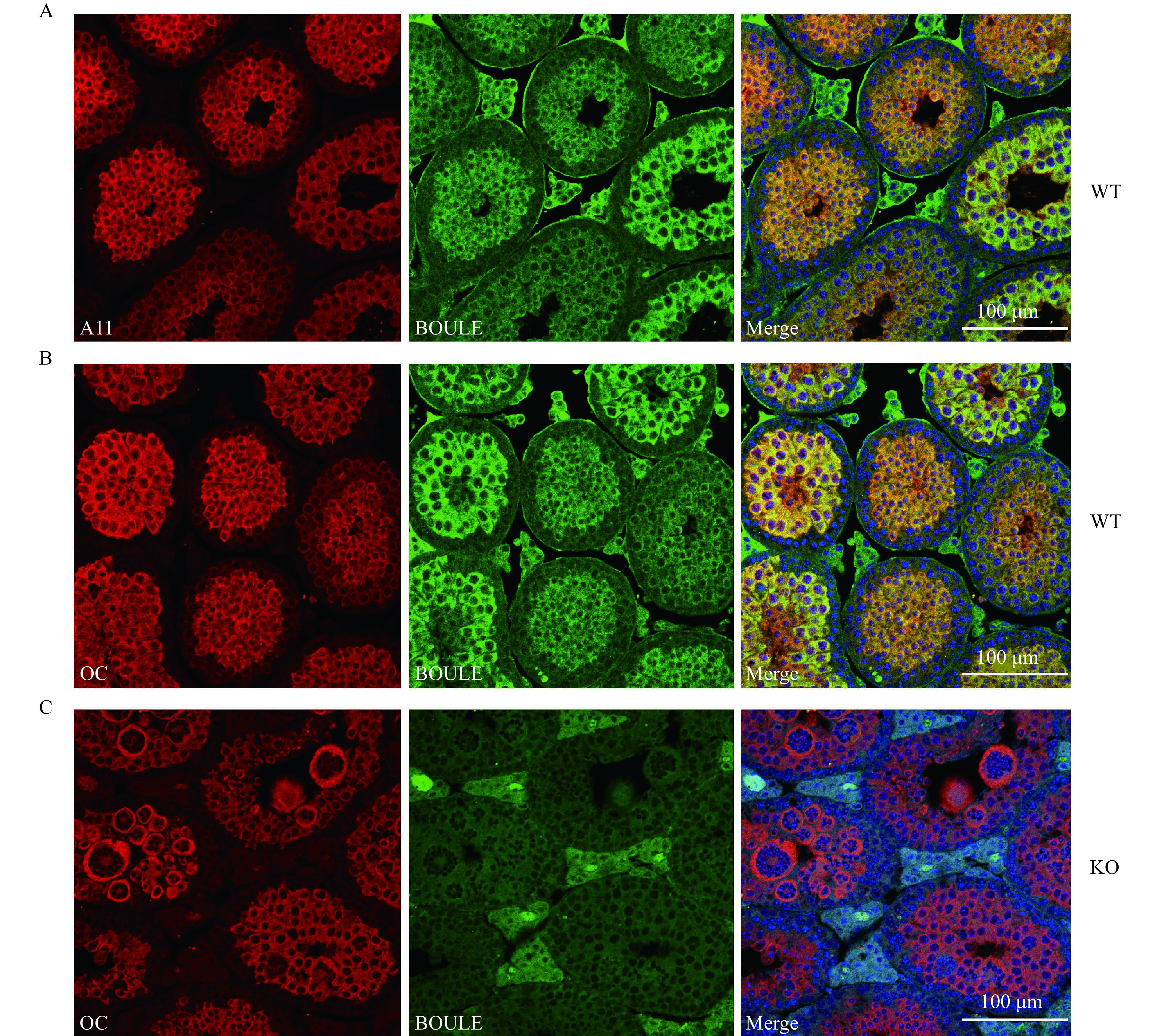
Mouse BOULE protein co-localized with amyloid structures during spermatogenesis.

We next investigated whether BOULE protein expression and aggregates expression occur during spermatogenesis by immunofluorescence staining of BOULE and with fibrillar and oligomeric amyloids extracted from mouse testis. Two conformation-specific antibodies A11 and OC, which recognize prefibrillar oligomeric and fibrillar forms of amyloid, respectively^[24]^, were used to visualize the expression of amyloids during spermatogenesis. Interestingly, BOULE signals colocalized with both A11 and OC signals in the testis (***Fig. 2A*** and ***B***). Specifically, BOULE and the aggregates are highly expressed in spermatocytes and round spermatids but appear absent in spermatogonia or elongating spermatids (***Fig. 2***). However, we also detected OC signals in *Boule* KO mice which suggests that BOULE is not the only protein in the testis that forms amyloid aggregates (***Fig. 2C***). Collectively, our data revealed that BOULE can form SDS-resistant high molecular amyloid-like aggregates in mouse testis.

### Key regions essential for the formation of BOULE aggregates

**Figure 3:**
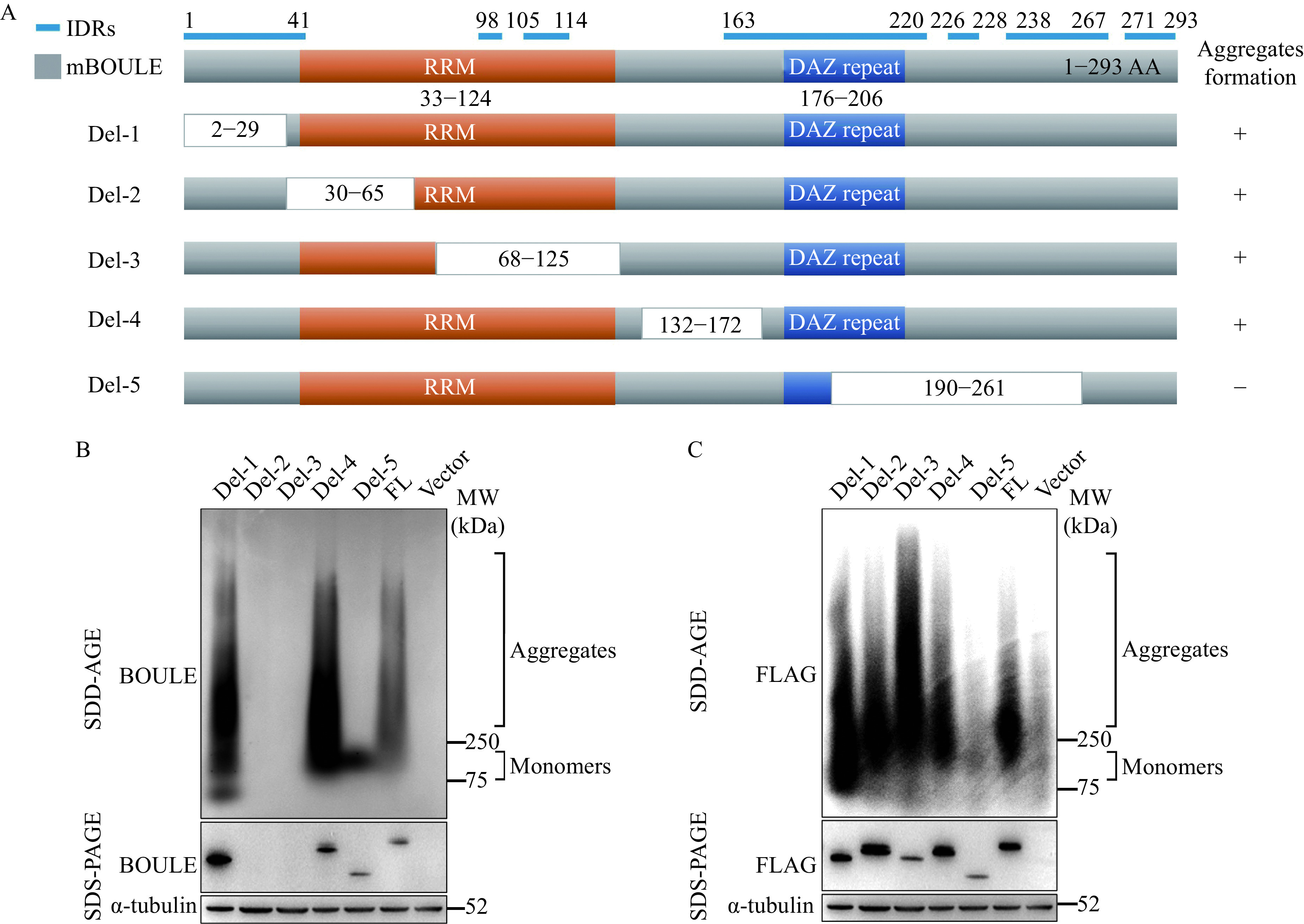
BOULE protein deletion constructs were used to map the region of BOULE essential for protein aggregation.

It is understood that proteins commonly contain low complexity domains (LCDs) or intrinsically disordered regions (IDRs) that can drive full-length proteins into liquid-liquid phase separation (LLPS) through weak multivalent interactions^[7,25–26]^. It is also held that this can promote the formation of aggregates. Phase transition or condensate could be early-stage of BOULE aggregate formation. Therefore, we investigated this scenario and found several IDRs in mouse BOULE protein *via* MobiDB (***Fig. 3A***). To determine which region of BOULE proteins is involved in aggregate formation, we constructed five different FLAG-tagged BOULE deletion constructs (Del-1, Del-2, Del-3, Del-4, and Del-5). Using SDD-AGE, we found those lacking Del-5 segment 5 (*i.e.*, amino acids 190–261) in BOULE protein failed to form aggregates (***Fig. 3B*** and ***C***). Therefore, amino acids 190 to 261 contain sequences which are vital when forming SDS-resistant aggregate structures (***Fig. 3B*** and ***C***).

**Figure 4:**
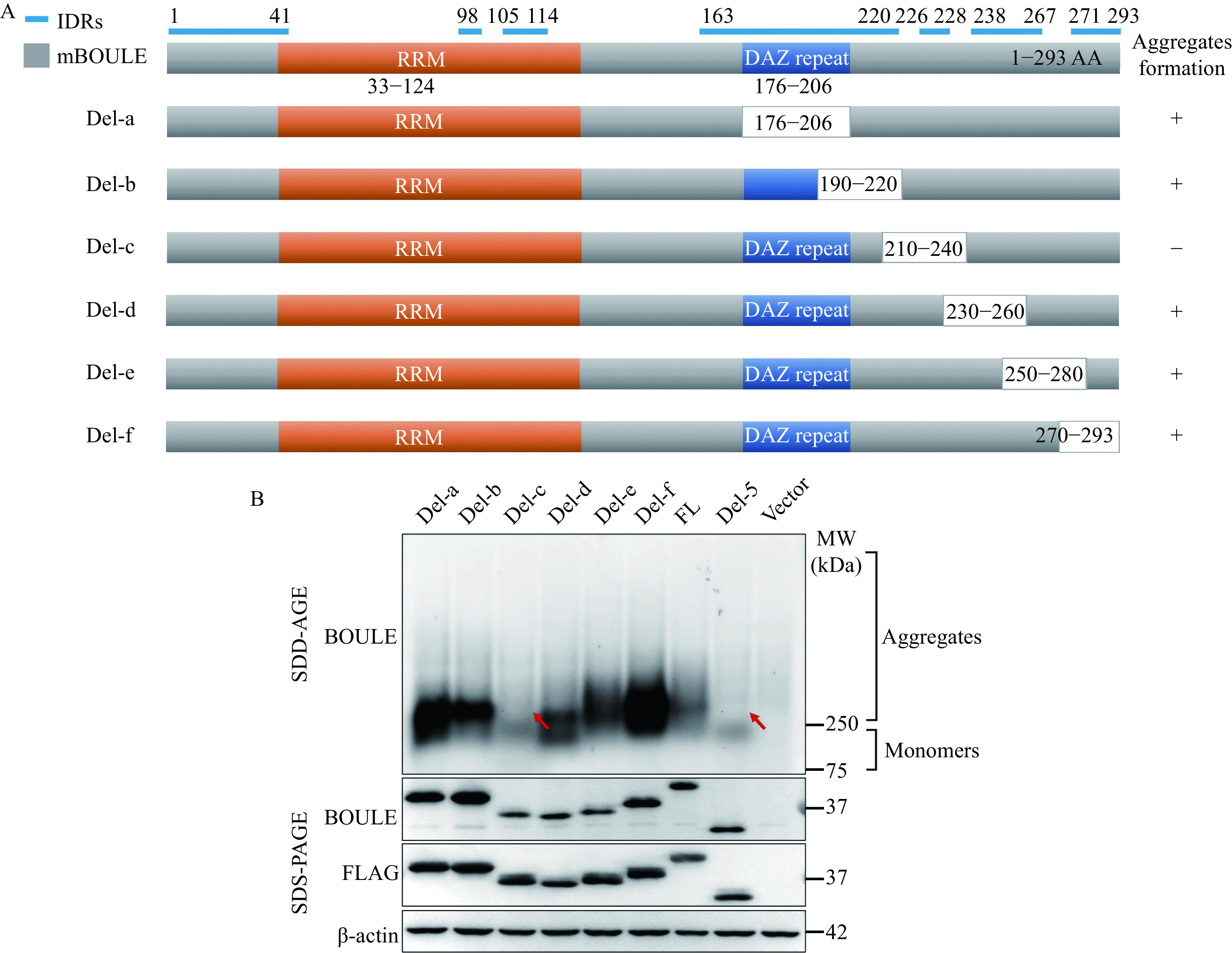
A 30-amino acid region downstream DAZ repeat is critical for BOULE protein aggregation.

To further specify the region, we constructed six smaller deletions of the segment 5 region (Del-a, -b, -c, -d, -e, -f). We found that removal of the Del-c region (amino acids 210–240) of BOULE protein creates a deficiency in aggregation-forming abilities (***Fig. 4B***). This enabled us to hone in on the BOULE region to understand the formation of BOULE aggregates to amino acids 210 to 240, consisting of VPQSPASSAPFLYLQPSEVIYQPVEIAQDGG. This small segment of peptides also contains sequences involved in an intrinsically disordered region (***Fig. 4A***) and is highly conserved in BOULE homologs of many mammalian species (data not shown). The generation of knock-out mutations which disrupt this region may provide insight into the function of BOULE aggregation in spermatogenesis.

#### BOULE aggregation was susceptible to heat shock

**Figure 5:**
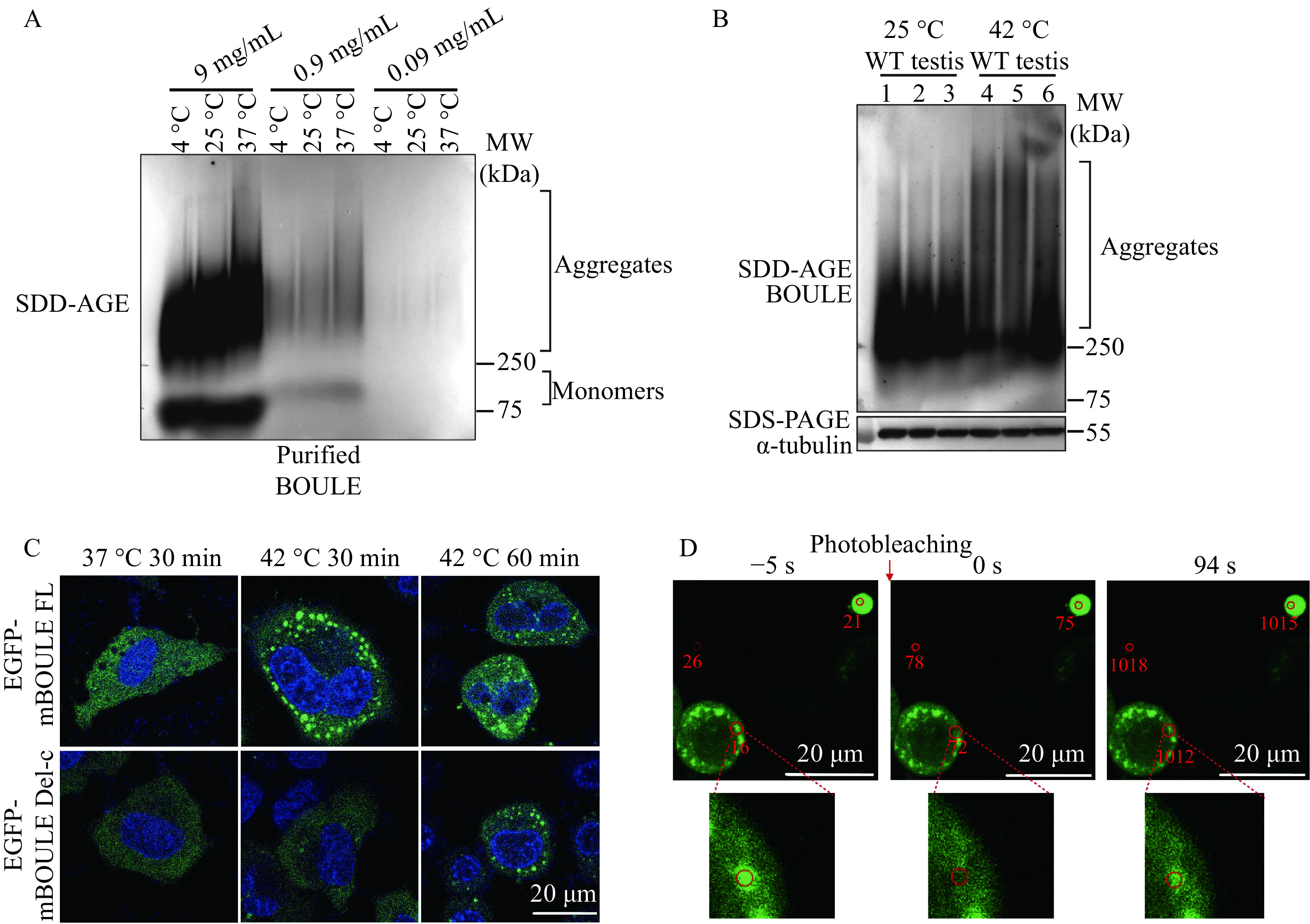
Effect of temperature on BOULE protein aggregation using purified BOULE protein, EGFP tagged BOULE expressing Hela cells, and mouse testes.

Since we found that BOULE aggregates in the testis and that 210–240 AA of BOULE protein is crucial for the aggregation state of BOULE, we investigated whether BOULE can aggregate *in vitro*. To answer this question, we expressed 6× His-tagged mouse BOULE full-length proteins in bacteria and purified the proteins (***Supplementary Fig. 1***, available online). Purified mouse BOULE incubated at different temperatures and different concentrations were analyzed by SDD-AGE assay to determine whether purified BOULE protein could form aggregates. We found that at 0.9 mg/mL (28 mmol/L) and higher, BOULE does clearly form protein aggregates. Higher temperatures promoted purified BOULE protein to form aggregates with high molecular weights (***Fig. 5A***), though at a concentration of 0.09 mg/mL (2.8 mmol/L) BOULE aggregation is still detectable. To exclude the potential impact of His-tag on BOULE protein aggregation property, we constructed N-terminal, C-terminal full-length BOULE protein together with *Boule* deletion construct (Del-c) and our data (***Supplementary Fig. 2***, available online) further supported that BOULE full-length protein formed aggregates.

We also examined the effect of heat stress on BOULE aggregate formation and found that heat shock treatment of mouse testes at 42 °C increased BOULE aggregation in mouse testes compared to non-heated, shocked mice (***Fig. 5B***). This is consistent with the previous report of DAZ family proteins in stress granules^[27]^.

Next, we constructed BOULE FL, BOULE protein 210–240 amino acids deletion construct (BOULE Del-c), and enhanced green fluorescent protein (EGFP) fusion constructs and transfected them into Hela cells to visualize their aggregation in the cells. We found that the FL-BOULE-EGFP protein phase-separated into droplets rapidly in fewer than 30 minutes after 42 °C heat shock. Yet, Delc-BOULE-EGFP only formed some droplets 60 minutes after heat shock treatment (***Fig. 5C***).

The FRAP method can be generally used to determine the dynamics and mobility of phase-separated liquid droplet or condensate in live cells. Moreover, if EGFP is fused to a protein, the mobility of the protein of interest can be calculated following the EGFP signal in the bleached area. We determined the mobility of FL-BOULE-EGFP liquid droplets in Hela cells by FRAP and found that the BOULE droplets exhibited a certain degree of recovery properties. This further suggests that the formation of BOULE droplets arises through phase separation (***Fig. 5D***).

### Identification of BOULE aggregates targets in mouse testes by eCLIP

**Figure 6:**
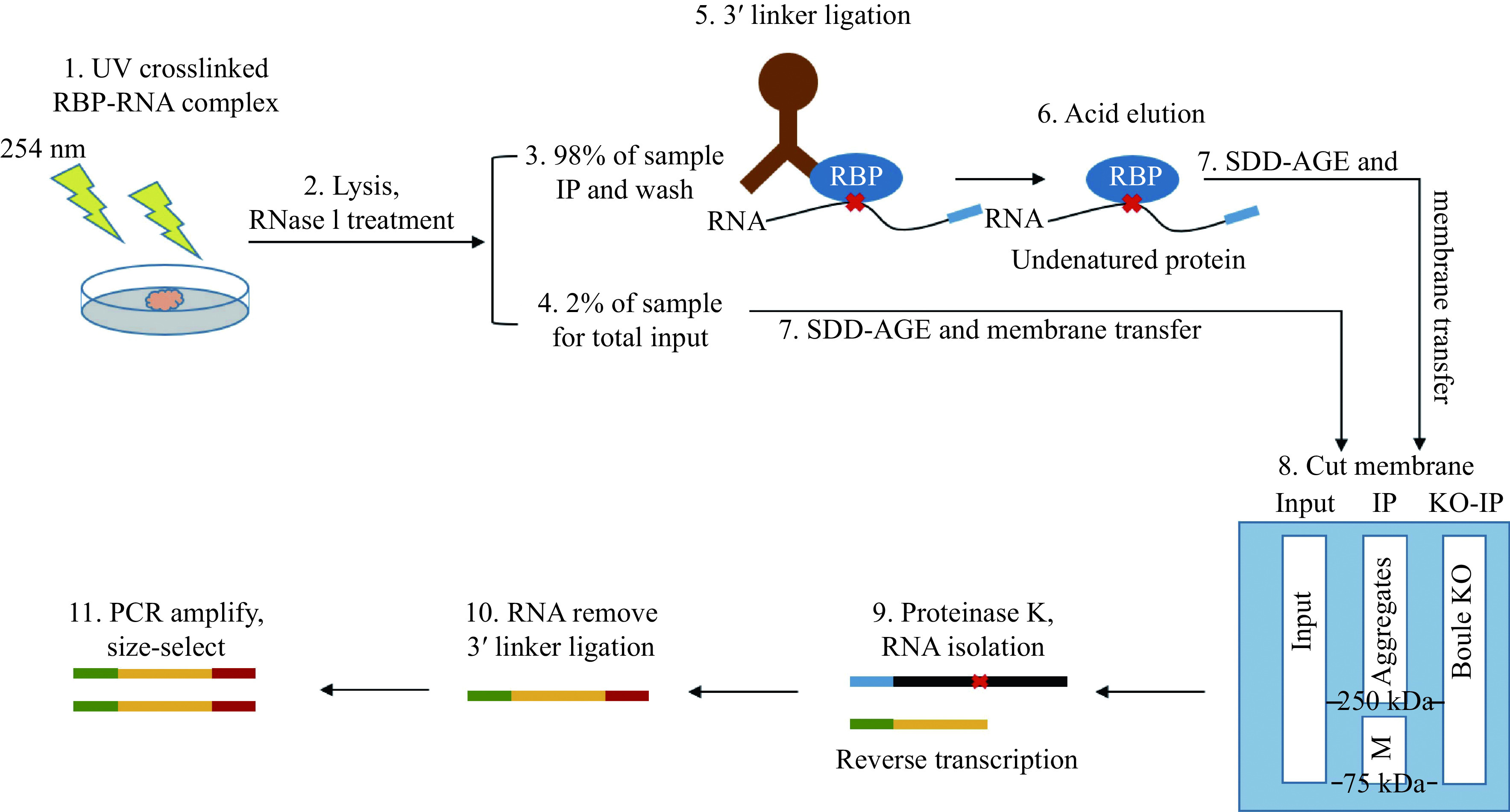
Schematic diagram of eCLIP experiment procedure.

**Figure 7:**
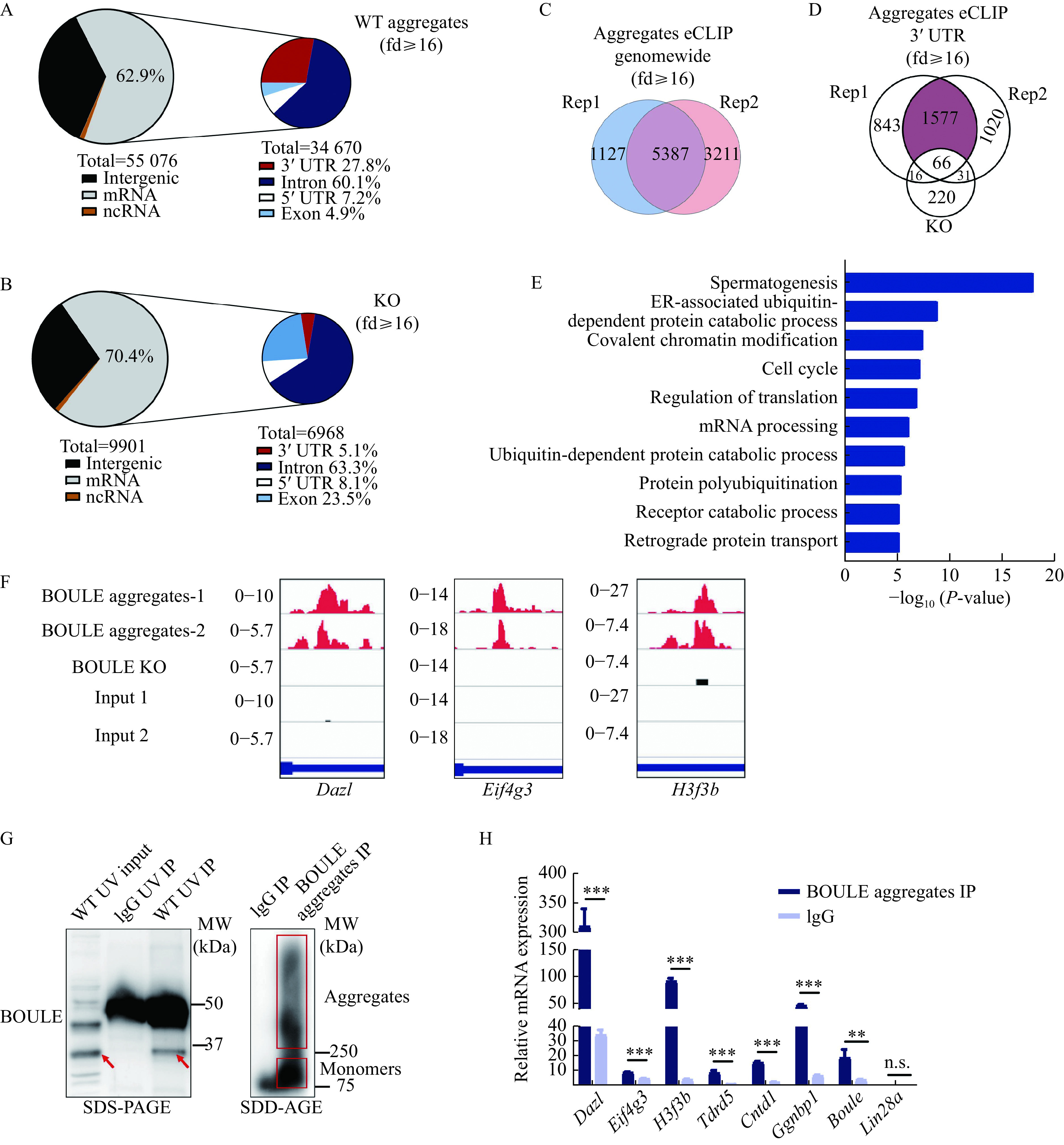
Identification of BOULE aggregates targets in mouse testes by eCLIP.

To shed light on how BOULE aggregates function during spermatogenesis, we performed an eCLIP protocol to identify transcriptome-wide RNA targets of the BOULE aggregates (***Fig. 6***). While eCLIP has been widely used in identifying RNA targets bound by RNA binding proteins, there is no report on using eCLIP to identify mRNAs bound by RNA binding protein aggregates^[28]^. Mouse testes were subjected to UV-mediated crosslinking, lysis, and treatment with a limited amount of RNase, followed by immunoprecipitation (IP) of protein-RNA complexes similar to standard eCLIP procedure. To avoid the influence of targets bound by the BOULE monomer, we excised the bands above 250 kDa to avoid any overlapping targets with monomer BOULE (***Fig. 6*** and ***Fig. 7G***). RNA targets from two replicates from BOULE aggregates eCLIP overlapped extensively supporting the reproducibility of the targets-identification. The eCLIP peaks of at least 16-fold enrichment over the input lane were considered as BOULE aggregate targets. The resulting eCLIP transcripts from two biological replicates were filtered and mapped individually and were then intersected to identify 5387 overlapping eCLIP genomic transcripts (***Fig. 7C***). Compared with the knock-out group, we find that the 3′ UTR of BOULE aggregates targets have the greatest change in the whole genome (***Fig. 7A*** and ***B***). We thus focused our analysis on mRNA targets with BOULE aggregates binding sites at the 3′ UTR region. After removing the influence of *Boule* knockout and monomer non-specific targets, we found that BOULE aggregates bind to 1577 transcripts in adult testis on their 3′ UTR (***Fig. 7D***). Those targets were significantly enriched in spermatogenesis pathways (classified by DAVID). Please see ***Fig. 7E*** for further details.

We further confirmed the quality and reproducibility of the two libraries by comparing the binding sites and peaks among the targets by Genome browser tracks (http://genome.ucsc.edu/). We observed a consistent peak distribution for the same target transcripts among the two libraries (***Fig. 7F***). To determine the validity of the BOULE aggregates target mRNAs identified by eCLIP, we randomly selected six targets from spermatogenesis pathways among the 1577 3′ UTR targets shared by two libraries and found they were all significantly enriched in BOULE aggregates IP. *Boule* is used as positive control, by contrast, non-targets such as*Lin28a* were not enriched (***Fig. 7G*** and ***H***). This suggested that the 1577 shared targets were reliable BOULE aggregates targets. Hence, we conclude that the BOULE aggregates specifically bind to a large number of transcripts, leading us to propose that BOULE aggregates may have a physiological function during sperm development.

## Discussion

In response to stimulus, cells constantly face the demanding task of promptly organizing various molecular reactions. To address this challenge, cells have evolved compartments which facilitate spatiotemporal control of biological reactions. Many membrane-less organelles, such as stress granules and P-bodies, dynamically assemble and disassemble in response to cellular stress and other stimuli^[29–30]^. A number of studies have identified many RNA-binding proteins, such as hnRNPA1, and TDP43, which contribute to this conditional cellular compartmentalization. These proteins commonly contain LCDs or IDRs that can drive full-length proteins into LLPS through weak multivalent interactions^[25–26]^. However, at high concentrations, many proteins form amyloid-like filaments consisting of repeated cross-beta strand elements^[31–32]^, which typically have been viewed as pathological entities associated with neurodegenerative disease and prionopathies^[33]^. Although, evidence increasingly indicates that most proteins can adopt the functional amyloid fold under certain conditions and that some amyloids carry-out non-pathologic biological roles.

When proteins aggregate, they usually form into an amyloid state consisting of cross β-sheet structure forming long fibrils. At the same time, amyloids also exhibit several other unusual properties, such as detergent and protease resistance, interaction with specific dyes, and the ability to induce the transition of some proteins from a soluble form to an aggregated one. These same features have also been demonstrated for the aggregates lacking cross-β structure, which are commonly called "amyloid-like". The conformational switch into an amyloid-like state is often mediated by multivalent interactions among proteins containing low-complexity regions, which are intrinsically disordered.

In this study, we found that the testicular-specific BOULE protein can form amyloid-like aggregation *in vitro* and *in vivo*, and BOULE aggregates showed identical developmental expression patterns (detected by SDD-AGE) with BOULE monomers (detected by SDS-PAGE). Through different BOULE protein deletion experiments, we found that the key region essential for the formation of BOULE aggregation was located with the peptide of #210-240AA, which happens to be located in the IDR region. The aggregation of BOULE was affected by temperature, when temperature increased the aggregation of BOULE protein increased significantly and rapidly. Interestingly, testicular BOULE proteins also undergo phase separation after heat shock, and FL-BOULE-EGFP droplets exhibit clear mobility after laser treatment, supporting BOULE protein undergoes aggregation. Phase transition may be one of the transient states during BOULE aggregation into amyloid-like state. As RNA binding proteins function through binding RNA targets, we are asking whether BOULE aggregates might bind to any RNAs. We also found that BOULE aggregates bind to a large number of spermatogenesis-related mRNAs by eCLIP, supporting potential functional roles of BOULE amyloid-like aggregates.

Amyloids are fibrous protein aggregates that are known for their roles in the etiology of neurodegenerative diseases. The formation of amyloid is a multi-step process in which proteins progress from soluble oligomers to fibrils. Proteins which form functional amyloids follow similar aggregation pathways to those that form pathological amyloids, including the development of cytotoxic intermediate oligomeric amyloids^[34]^. Of the functional amyloids, many proteins transition quite rapidly under controlled cellular conditions from monomeric to mature amyloid forms, possibly as a means to avoid cytotoxic intermediates^[3]^. Indeed, unlike pathological amyloids such as α-synuclein, which require days to weeks to form mature amyloid fibrils *in vitro*, proteins forming functional amyloids often do so within minutes to hours. Our results showed that the kinetics of BOULE amyloidogenesis is similar to consistent with other proteins that form functional amyloids by showing rapid rates of amyloid formation *in vitro*. Other reproductive functional amyloids also exhibit fast rates of the amyloid formation including Xvelo, the amyloid component of Balbiani bodies, which transitioned into a fibrillar matrix after 12–24 hours^[4–35]^. Similarly, fibrils were detected in recombinant Rim4 after overnight incubation^[1]^.

Previous studies have shown that budding yeast builds massive RNA-binding protein structures that exhibit biochemical properties of amyloid to regulate translation during meiosis. Reported existence of amyloid-like assemblies in mouse and frog meiosis led to the hypothesis that amyloid-like assemblies are a conserved feature of gametogenesis^[1]^. Our work on BOULE aggregate formation in the testis supports the evolutionary conservation of functional amyloid-like RNA binding proteins during mammalian spermatogenesis. Further study on DAZ family proteins' aggregate formation and their RNA target expression may elaborate our understanding of the function of the amyloid-like states in DAZ proteins. Establishing mouse models disrupting aggregation without impacting protein expression of BOULE will be a key to delineating the function of BOULE or DAZL amyloid-like aggregates on spermatogenesis. In combination with eCLIP target comparisons between aggregates and monomers, we should also investigate the molecular mechanisms involved in BOULE protein amyloid-like aggregates function to promote sperm development. We also need clues about what creates the conservation of DAZ family proteins in metazoan gametogenesis.
